# A Decision of the Court of Appeal and Its Effects on the Right to Benefits

**Published:** 1916-05-20

**Authors:** 


### NATIONAL INSURANCE ACT DEVELOPMENTS.

### A Decision of the Court of Appeal and its Effects on the Right to Benefits.

Considering the widespread application of the National Insurance Acts there has been singularly little litigation in the Courts as the result of their enactment. There has been nothing like the litigation, for instance, which has arisen out of the Workmen's Compensation and Employers' Liability Acts. This absence of litigation is, of course, due to the special provisions that were inserted in the principal Act, which provide that disputes shall be settled on specific and well-defined lines, into the machinery of which arbitration by specially constituted authorities, with the right of appeal to the Insurance Commissioners, largely enters.

Now, however, a test case of first-rate importance has reached the Court of Appeal, and the unanimous decision of that Court appears to decide finally—at least until the introduction of further legislation—a question which materially affects the right of married women to benefits under the Acts. It is even possible that the effect of the judgment will restrict the rights to benefit not only of married women, but also of all insured persons, either men or women.

### A Claim for Maternity Benefit.

The circumstances of the case, which is known by the title *Davidson* v. *New Tabernacle* (*Old Street Congregational*) *Approved Society*, are interesting quite apart from the general question which arises therefrom. It will, therefore, be as well to state the salient features of the case, thus showing how the general question came up for decision, before proceeding to deal with the judgment and its effects.

Ruth Davidson, who was insured in the New Tabernacle Approved Society, was engaged in pottery works, lifting and carrying clay from outside for use in the works. She was employed under a contract of service, and continued to be so employed up to August 4, 1913. On that day she relinquished her work for the purpose of being married, and was in fact married on the following day. In the December following a child was born, and thereupon a claim was made upon her society for the payment of maternity benefit. This claim was resisted, and there was arbitration, the arbitrator giving his award in favour of the society. From that arbitration there was a re-hearing before the Insurance Commissioners, who decided in favour of Ruth Davidson. In normal circumstances their decision would have been final, but as their award was made in the form of a special case the society again appealed, and the matter was heard by Mr. Justice Atkin in the King's Bench Division. The judgment of Mr. Justice Atkin was also in favour of Ruth Davidson, but, still unsatisfied, the society entered an appeal, with the result that in the Court of Appeal the case was decided in their favour.

The Court of Appeal was particularly strong, being composed of Lord Justice Swinfen Eady, Lord Justice Pickford, and Lord Justice Bankes. Each of their Lordships delivered an independent judgment setting forth the grounds for his decision; and, as their awards were unanimously in favour of the society and against the decision of the Insurance Commissioners, there does not appear to be any prospect of the case being carried to the House of Lords.

### The Matter in Dispute.

The dispute as to the right of the woman to the maternity benefit turned on the question as to whether, on the date of her confinement, she was or was not an insured person.

Up to the day upon which she relinquished her work for the purpose of being married there is no dispute that she was undoubtedly insured and entitled to the ordinary benefits. In point of fact she actually relinquished her employment because she was not in a fit state of health to carry out the heavy work which that employment involved. She intended, after her recovery from her confinement, to return to work; and, although there was no agreement on the part of her former employers to re-engage her, it was contended that she would not have experienced any difficulty in securing similar employment to that upon which she was engaged prior to her marriage if her health had permitted her to undertake it. The evidence showed that she did not actually resume her work. It was on these grounds—namely, that the woman left her work because she was unable to perform the duties, and that she had the *bona-fide* intention to return, that the Insurance Commissioners decided in her favour. Their decision at first sight seems reasonable enough.

Lord Justice Swinfen Eady called attention in his judgment, however, to the express provision contained in Section 44 of the Act of 1911, which provides that "where a woman, who has before marriage been an insured person, marries, she shall be suspended from receiving the ordinary benefits . . . until the death of her husband." To this direct statement, which would *prima facie* have suspended the woman from the right to benefit, there is a proviso under which it was contended that the case would come. The proviso is to the effect: "Provided that, where a woman who has been employed within the meaning of this part of the Act before marriage, proves that she continues to be so employed after marriage, she shall not be so suspended so long as she continues to be so employed."

His Lordship ruled that the language here used means that the woman has to prove that after her marriage she has actually been or continued to be employed under a contract of service. If instead of relinquishing her work the woman had been granted a holiday by her employers, on the understanding that her post would be kept open for her until after her marriage and recovery from her confinement, the case might have been different. In those circumstances it might have been contended successfully that, though she did no actual work, she still continued to be employed under a contract of service, and thus have been able to claim the privilege extended by Section 79 of the Act, which provides that for certain purposes "a person whose normal occupation is employment within the meaning of the Act shall . . . be deemed to continue to be an employed contributor notwithstanding that he is temporarily unemployed."

In other words, his Lordship held that it was not sufficient for the woman to state that she had a *bona-fide* intention to continue in employment; she had to prove that she had actually been employed under a contract of service.

### The Application of the Judgment.

The full significance of the judgment may escape attention, except upon closer observation. In effect it means this, that any woman, who before marriage was an employed contributor, ceases *ipso facto* to be entitled to the ordinary benefits immediately upon her marriage. So long, and only so long, as she continues to be employed after her marriage she is entitled to resume the right to benefit, but immediately she becomes unemployed, even if only temporarily, she forthwith loses her right to receive benefits.

If this decision is to apply in all cases it is easy to show that it will frequently entail considerable hardship. Take the case of a woman who is employed in a factory by the day or week or on piece-work. She was married since she first became insured. She does not feel quite up to her work and stays away. In a few days she becomes worse and goes to her panel doctor, who advises that she should go to bed, and specifies the disease from which she is suffering. She thereupon claims sickness benefit from her society. If the society is fortified by the judgment to resist the claim on the ground that the woman is no longer actually employed, then it is clear that a grave injustice will be done. It is clear that a person cannot actually be employed and at the same time be in a position to claim sickness benefit on the ground of inability to work.

In these circumstances it is not surprising, perhaps, that Lord Justice Swinfen Eady should state that if it were not for the express language of the section of the Act "one would be glad to have arrived at a different conclusion, having regard to the facts of the case," and we presume that some steps will be taken to ensure the right to benefits, for which contributions have been paid, in the Amendment Act, which should come forward in the near future as the result of the deliberations of the Committee of Inquiry.

We suggested at the outset that the judgment might conceivably be made to apply to men and unmarried women as well as to married women. This possibility arises through the stress which all three of their Lordships placed upon the interpretation of the words "so employed," meaning, as they stated, "employment under a contract of service," and the importance they attached to the words signifying the purposes for which persons whose normal occupation is employment within the meaning of the Act shall be deemed to continue to be employed contributors although temporarily unemployed. These purposes are for "reckoning the number and rate of contributions." Nothing whatever is prescribed in that section as to persons being deemed to continue to be entitled to benefits though temporarily unemployed, though doubtless it was intended that they should be.

In view, therefore, of the great importance attached by their Lordships to the precise language employed, it would appear to be necessary to put the matter beyond dispute by incorporating in an Amendment Act appropriate words which shall give the right, beyond all question, to benefits for which contributions have been paid, so long as the contributor has not changed his status so as to be no longer entitled to continue such contributions.

